# Longitudinal associations of DNA-methylation of *OXT*, *SLC6A4* and *NR3C1* genes with the treatment response in patients with depression

**DOI:** 10.1038/s41598-026-57641-9

**Published:** 2026-06-16

**Authors:** Simon Sanwald, Thomas Kammer, Bernhard J. Connemann, Christian Montag, Markus Kiefer

**Affiliations:** 1 Department of Psychiatry and Psychotherapy III, Ulm University, Ulm, Germany; 2 Centre for Cognitive and Brain Sciences, Institute of Collaborative Innovation, University Macau, Macau SAR, China; 3 Department of Psychology, Faculty of Social Sciences, University of Macau, Macau SAR, China; 4 Department of Computer and Information Science, Faculty of Science and Technology, University of Macau, Macau SAR, China

**Keywords:** Epigenetics, Depression severity, Longitudinal, Methylation change, mRNA, Blood cell composition, Biomarkers, Diseases, Genetics, Medical research, Neuroscience

## Abstract

Altered methylation of genes implicated in depression has been proposed as a biomarker for treatment response. Yet, replication remains inconsistent while methodological concerns have grown. In a matched case control study, we investigated DNA-methylation of the *NR3C1* (glucocorticoid receptor), *SLC6A4* (serotonin transporter), and *OXT* (oxytocin) genes in peripheral blood of patients with depression (*N* = 66) and healthy controls (*N* = 66) at baseline and after two weeks of inpatient treatment. We tested group differences, convergence over time, associations with changes in depression severity, and relations to mRNA abundance. At baseline, *OXT* methylation was significantly lower in patients, but this effect disappeared when adjusting for neutrophil and lymphocyte abundance, highlighting the impact of cellular composition on methylation measures. Despite significant decrease in depression severity, methylation changes in none of the candidate genes were associated with treatment response. In healthy controls, two *OXT* CpG sites showed negative associations with mRNA abundance, which were absent in patients. These findings emphasize the importance of rigorous control of confounders, especially blood cell composition, in psychiatric epigenetics. Our results add to accumulating null findings for stress- and serotonergic-related genes and caution against overinterpreting peripheral methylation signals as biomarkers for depression.

**Supplementary Information:**

The online version contains supplementary material available at 10.1038/s41598-026-57641-9.

## Introduction

Major Depressive Disorder (MDD) is an affective disorder characterized by episodes of depressive mood and/or loss of pleasure in combination with issues concentrating, disturbances in sleep and appetite affecting an individual’s ability to function^1^. Because of the episodic nature of the disorder, an objective marker of depression that not only predisposes an individual for depression development but also changes along with symptoms of the disorder would be an invaluable target for understanding depression and for the development of new treatment options. With the discovery of the plastic and modifiable nature of epigenetics in animal studies^2^, a new hope was sparked that among these processes could be the objective marker that has long been searched for.

Epigenetics involves the biochemical modification of DNA or associated proteins that leads to the structural adaption of chromosomal regions, serving to register or signal specific activity states without altering the underlying DNA-sequence^3^. The most investigated epigenetic process is DNA methylation which is mostly defined as the addition of a methyl group to the 5’position of cytosines in cytosine-guanine dinucleotides (CpG-sites). This DNA-modification can be – depending on the specific genomic location – associated with repression of gene transcription by blocking transcription factors^4^.

There are two approaches for studying epigenetic mechanisms in psychiatry: Epigenome-wide associations studies (EWAS)^5^ offer an unbiased, hypothesis-free exploration of the methylome, essential for identifying novel pathways to a disorder’s pathophysiology. By surveying a vast amount of CpG sites simultaneously, EWAS are able to capture systemic epigenetic signatures that candidate gene models might overlook. Moreover, EWAS provide a more comprehensive understanding of the complex regulatory landscape underlying psychiatric conditions. In contrast to EWAS, candidate gene approaches^6,7^ offer superior read depth and coverage of specific genomic loci, not only allowing for initial discovery but also for targeted validation of findings from EWAS. By focusing on a limited number of CpG sites, they substantially mitigate the burden of multiple testing, thereby increasing statistical power. Last, the candidate gene approach facilitates hypothesis-driven research, allowing for validation of mechanisms derived from established pathophysiological models or preclinical evidence. The current study is a candidate gene study on three genes implicated in theories of depression development^8–10^:

First studies investigating the association of early life stress and depression development later in life focused on methylation of the glucocorticoid receptor gene (*NR3C1*)^2,11^. Glucocorticoid receptors in the hippocampus are relevant for negative feedback and thus for dampening of the stress response^12^. Altered abundance of glucocorticoid receptors is in line with the observation of altered stress responses and hypercortisolism in individuals suffering from depression^13^. The aforementioned early methylation studies postulated licking and grooming behavior of dams to program the *NR3C1* gene of pups by changing its methylation status, which resulted in altered stress responses and depressive symptoms. Methylation of *NR3C1* in rat pups was reversible and reversing the methylation normalized stress responses and reduced depressive symptoms^2,11^. This led to the examination of *NR3C1* methylation but also other genes important for the functioning of systems assumed to be closely related to depression in human samples^14^. Amongst the investigated systems are the serotonin and the oxytocin system. Investigated candidate genes are for example the serotonin transporter gene (*SLC6A4*) and the gene coding for oxytocin (*OXT*)^15–17^. While the association of the serotonin system with depression is obvious from the main pharmacological treatment options for depression, i.e., selective serotonin reuptake inhibitors (SSRIs) or even recently re-evaluated classical psychedelics, the early monoamine deficiency hypothesis is long considered too simplistic, which is why there are efforts to formulate an updated version taking into account the importance of serotonin for depression development and course without the simplistic causal notions of earlier versions^10,18^. Oxytocin on the other hand has been shown to have stress buffering properties, while being implicated in social behavior and adaptive processes throughout the lifespan – i.e., oxytocin is thought to be associated with resilience or to buffer depressive symptoms^19,20^. However, when transferred to human samples, mixed results were reported regarding the associations between methylation of these genes and depression: With respect to candidate gene studies, a recent review concluded that *NR3C1* was the gene studied the most, however there have been inconsistent results, i.e., there are reports of hypomethylation as well as hypermethylation in depression and also null findings^7^. While for *SLC6A4* the majority of studies reported hypermethylation in depression, there are also conflicting results^7^. As for *OXT*, studies from our group repeatedly showed hypomethylation in patients suffering from depression compared to healthy controls^15,16,21^. A further study from a different group showed hypermethylation of *OXT* in maltreated children and adolescents – childhood maltreatment is a well-established risk factor for depression development^13^– compared to healthy controls^22^. However, methylation studies oftentimes did not have longitudinal data, did not adjust for blood cell composition or associations with mRNA levels. Heterogenic results of studies examining methylation of candidate genes were explained by the following challenges among others^23^: DNA-methylation is studied by collecting peripheral tissue samples and not directly in the brain, there are mostly cross-sectional studies with lots of confounding factors like cell type composition of blood samples, substance use or other environmental factors. In addition, the comparability of study suffers from very heterogeneous samples, e.g., high power comes with the cost of insufficiently characterized or subclinical samples.

However, a few studies investigated longitudinal changes in DNA-methylation, mostly in the context of different treatment options for mental disorders like post-traumatic stress disorder (PTSD), depression, borderline personality disorder (BPD), panic disorder and alcohol use disorder^24–31^. While Carleial and colleagues^28^ found that PTSD-specific psychotherapeutic treatment significantly predicted methylation change from baseline to follow up in four CpG sites of four different genes associated with memory formation in an epigenome wide methylation study (EWAS), Vinkers and colleagues^27^ showed successful trauma therapy to be associated with methylation change comparable to the methylation change of trauma-exposed controls that did not develop a PTSD or receive treatment. The third EWAS on PTSD treatment and methylation change showed several genes’ methylation to change with severity of PTSD, one of it being FKBP5 encoding for a glucocorticoid receptor co-chaperon. Carvalho Silva^31^ and colleagues found that a trauma-specific psychotherapy for patients with treatment resistant depression was associated with 110 differentially methylated regions mainly in a different set of genes associated with inflammatory processes in an EWAS. The investigation of methylation changes during confrontation therapy and over the course of cognitive behavioral therapy in panic disorder revealed changes in several CpG sites, one site being within the serotonin transporter 3 A, with either confrontation or therapy course^26^. Quevedo and colleagues^24^ found the *FKBP5* gene to show a decrease in methylation in a very small sample of patients with borderline personality disorder as a function of trauma and responding to psychotherapy – note that the found three-way interaction should be interpreted with caution since sample size was not as calculated a-priori due to the COVID-19 pandemic. The examination of associations between electroconvulsive therapy and methylation change revealed methylation changes in several CpG sites one of it being in the FKBP5 gene in an EWAS conducted in a sample of *n* = 34 depressed patients^25^.

These studies mainly examined epigenetic changes comparing baseline measures to measurements after completion of the respective treatment, i.e., after a time period of at least one month. However, changes in depressive symptoms already occur in early treatment phases^32^. In addition, changes of depressive symptoms as soon as two-weeks after the beginning of treatment with antidepressants are predictive for treatment outcome^33^. Therefore, in patients diagnosed with depression methylation should also show plasticity in initial treatment periods alongside changes in depressive symptoms in order to be a promising candidate mechanism for future interventions.

In summary, an investigation of the associations between changes in DNA-methylation of *NR3C1*, *SLC6A4* or *OXT* and changes in depression severity during the first weeks of treatment is important to elucidate whether methylation of these genes is related to the treatment response. Further, examination of functional relevance regarding gene transcription in combination with the analysis of longitudinal associations of methylation and depression independent of potentially confounding factors like substance use, medication, body mass index (BMI) or blood cell composition is valuable to assess the usefulness of methylation of target genes in peripheral blood samples as biomarkers for depression.

Therefore, with this study (study protocol preregistered at https://osf.io/rwz2p), we address three research questions:

1. Is there a group difference in methylation between MDD patients and controls independent of substance use, medication and blood cell composition?
2. Does this group difference in methylation between MDD patients and controls get smaller after two weeks of treatment?
3. Is the change in methylation of *NR3C1*, *SLC6A4* and *OXT* after a two-week (treatment) period associated with a change in depression severity?
4. Is methylation of these candidate genes associated with mRNA abundance at the first measurement point?

## Methods

### Participants

Data of *N* = 87 inpatients suffering from MDD and *N* = 132 healthy controls (HC) was collected. Depressed inpatients were recruited at the Department of Psychiatry and Psychotherapy III. HCs were recruited via online and offline advertisement. 16 MDD patients were excluded because they were later during inpatient treatment diagnosed with a diagnosis other than MDD – note that comorbidity of anxiety disorders was not an exclusion criterion because of the high comorbidity rates of these disorders. 15 HCs were excluded due to a suspected past or present mental disorder on the basis of interviews using Mini-DIPS and SCID5-PD^1,34^ Thus, *N* = 71 patients were matched to *N* = 117 HCs by means of propensity score matching (body mass index (BMI), alcohol consumption and age) and exact matching (sex), resulting in a final sample of *N* = 66 MDD patients (a description of depression history is in Table 1; more details on treatments administered can be found in Table S1) and *N* = 66 HCs. This is 10% more than the minimum sample size based on the a-priori power analysis in our pre-registration (https://osf.io/rwz2p) considering missing data due to failure of methylation analysis for one of the three genes. However, the group difference we predicted at baseline in our first hypothesis requires a minimum sample size of *N* = 85 individuals per group to obtain a power of *1-β* = 0.85 based on an preregistered a-priori power analysis (https://osf.io/gdc54). Since this analysis was performed based on our findings regarding *OXT*^15,16^ and the effects reported for *NR3C1* and *SLC6A4* are small and heterogeneous as described above^7^, we used a matched sample of *N* = 95 individuals per group in order to account for missing data in any of the three genes and blood cell count. Since we collected data of *N* = 71 patients for the longitudinal study (after exclusions mentioned above), we investigated additional patients from our previous study, i.e., data overlapped with data of our previous study^15^(longitudinal sample: before matching overlap was 24, in the final longitudinal sample there were 14 overlapping patients; cross-sectional sample: before matching overlap was 40, in the final cross-sectional sample there were 34 overlapping patients). Analyses without overlapping patients are in the supplement (Tables S4–S7). Results obtained without additional patients were similar to the ones presented below. Participants were selected based on the recency of data collection, the presence of t1 data and the feasibility of matching HCs to MDD patients with respect to the above-mentioned covariates. Matching was implemented as described in the statistical analysis section. Note that we used an identical matching procedure as for the longitudinal sample, which is described below and analyzed the same CpG units for the cross-sectional and longitudinal analyses.

All procedures performed in this study were approved by the ethics committee of Ulm University, Ulm, Germany (approval date: 19th June 2023, number: 25/18) and all research was performed in accordance with relevant guidelines/regulations of the ethics committee of Ulm University and the Declaration of Helsinki. Written voluntary informed consent of all participants was obtained after the nature of the procedures had been fully explained.

### Questionnaires

Severity of depressive symptoms was explored by using the Beck Depression Inventory (German version, BDI-II)^35^. This self-assessment scale comprises 21 items with ratings from 0 (not at all) to 3 (very intense) indicating symptom complaints. A total of 63 points can be reached. Scores can be interpreted as follows: 0–8: no depression; 9–19 mild depressive syndrome; 20–28: moderate depressive syndrome; 29–63: severe depressive syndrome. Internal consistency was excellent in the group of individuals suffering from depression with α = 0.92 and good in the group of healthy controls, α = 0.88.

### Methylation analysis

Methylation of target genes was quantified by SEQ-IT GmbH & Co. KG (Kaiserslautern, Germany). Analyses were conducted by means of the Sequenom Epityper MassArray System (San Diego, CA, USA). All steps of the EpiTYPER assay were performed under routine conditions: Initially, DNA from peripheral blood was bisulfite treated (EZ DNA Methylation-Lightning Kit (CE-IVD); Zymo research, Freiburg, Germany). Then, amplicons for the CpG-rich regions in the *OXT*, *NR3C1* and *SLC6A4* promoter were designed using Agena’s EpiDESIGNER software (*OXT*: chr20:3,071,425-3,071,795; *NR3C1*: amplicon 1: chr5:143403938–143404340; amplicon 2: chr5:143403936–143404097; *SLC6A4*: chr17:30235345–30236068; amplicon 1: chr17:30235345–30235765; amplicon 2: chr17:30235734–30236068; hg38). After PCR amplification was performed, RNA was transcribed in-vitro. Subsequently base-specific cleavage using RNase A was performed, which resulted in fragmented RNA molecules of identical length but differing in their nucleotide composition due to bisulfite treatment. Probes were then processed on a MALDI-TOF platform (Agena; MassArray 4). Following preprocessing of the resulting data with the EpiTYPER Analyser, methylation status was quantified by analyzing the mass spectra. In line with the EpiTYPER protocol, an uncertainty threshold of 0.1 was used and all methylation values with an estimated error greater than the uncertainty threshold were excluded from further analyses. CpG units were included in the analysis only if methylation values were successfully obtained in at least 70% of samples (call rate ≥ 0.7).

### mRNA analysis

Expression analysis was performed for the first measurement point (t0) by SEQ-IT GmbH & Co. KG (Kaiserslautern, Germany). RNA was manually extracted in accordance with manufacturer’s instructions using the PreAnalytiX^®^ PAXgene^®^ Blood RNA Kit (IVD) (PreAnalytiX, Hombrechtikon, Switzerland). Quantity and quality of extracted RNA samples was measured photometrically using the BioTek Epoch Microplate Spectrophotometer (Agilent, Santa Clara, CA, USA) and adjusted to 50 ng/µl. In order to prevent RNA degradation and to remove DNA, extracted probes were treated according to manufacturer’s instruction with the recombinant ribonuclease-inhibitor RNaseOUT as well as DNase I (Invitrogen, Thermo Fisher Scientific, Waltham, MA, USA). Reverse transcription from RNA to cDNA was performed using SuperScript™ II reverse transcriptase in combination with Random-Hexamere-Primers (Invitrogen, Thermo Fisher Scientific, Waltham, MA, USA) in line with the manufacturer’s protocol. RNA mass was 650 ng absolute (13 µl à 50 ng/µl). Primers and probes for expression analysis (qPCR) were from TIB MOLBIOL (TIB MOLBIOL Syntheselabor GmbH, Berlin, Germany):

*NR3C1*:

*NR3C1* forward ATGATTGCATCATCGATAAAATTCG

*NR3C1* reverse TCCAACAGTGACACCAGGGTA

*NR3C1* probe 6FAM-TCTTGTGAGACTCCTGTAGTGGCC-BBQ

*OXT*:

*OXT* forward CGCAAGTGCCTCCCCT

*OXT* reverse CCTTCTGGCCGGACTGG

*OXT* probe 6FAM-AGGTAGTTCTCCTCCTGGCAGCG-BBQ

*SLC6A4*:

*SLC6A4* forward GGACGTGAAGGAAATGCTCG

*SLC6A4* reverse TGATGATCAACCGATAAGCTATATATGT

*SLC6A4* probe 6FAM-ATTTGCAGTTTTCTGATGAGCCCG-BBQ

*UBC* (Housekeeping Gene 1):

*UBC* forward GTCGCAGCCGGGATTT

*UBC* reverse TGCATTGTCAAGTGACGATCA

*UBC* probe 6FAM-AGCGATCCACAAACAAGAACTGCG-BBQ

*YWHAZ* (Housekeeping Gene 2):

*YWHAZ* forward TAGGTCATCTTGGAGGGTCGT

*YWHAZ* reverse ATCCCTTTCTTGTCATCACCA

*YWHAZ* probe 6FAM-TGTGAAGCATTGGGGATCAAGAACT-BBQ

Housekeeping genes were selected based on Vandesompele et al.^36^. qPCR was performed in duplicates using Hot Start Taq Probe qPCR Mix (ROX) (Axon Labortechnik GmbH, Kaiserslautern, Germany). Measurement was performed in duplicates on a LightCycler^®^ 480 Instrument II (Roche Diagnostics International AG, Rotkreuz, Switzerland). Cq values were obtained using the Second Derivative Maximum Method with Gene Maximization Set-up using two Inter-Run Calibrators^37^. Measures were analyzed with qbase + in accordance with Hellemans et al.^37^.

### Blood cell count

Hematological measurements were performed at Universitätsklinikum Ulm using a Sysmex XN-9100/Sysmex XN-1000 analyzer (Sysmex Corporation, Kobe, Japan). Cellular differentiation was conducted by fluorescence flow cytometry employing fluorescent dyes in combination with semiconductor laser technology, enabling leukocyte differentiation. Relative abundance was provided by the Sysmex-Middleware Extended IPU (Sysmex Corporation, Kobe, Japan). Absolute abundance of leukocytes was calculated from relative measures of different leukocyte types and is given in Giga/l.

### Statistical analysis

Data were analyzed using R statistics version 4.4.0^38^ in the RStudio environment^39^ using the packages *MatchIt*^40^, *lme4*^41^, *lmerTest*^42^, *ggplot2*^43^, *influence.ME*^44^ and *ggeffects*^45^. First, data was matched using a propensity score approach with the nearest neighbor method in case of age, alcohol consumption and body mass index (BMI) and exact matching in case of sex. Since patients smoked a significantly higher number of cigarettes per day, we could not match groups with respect to nicotine consumption. Since we were interested in potential diagnostic biomarkers of MDD in the cross-sectional analyses, we excluded all CpG-units that were significantly associated with nicotine consumption in either group, and we excluded all CpG-units that were associated with medication in the group of inpatients. In all analyses with longitudinal data, we included nicotine consumption and medication (dose equivalents for current antidepressants (weighted mean dose/fluoxetin40 mg)^46^ and neuroleptics (weighted mean dose/chlorpromazine100 mg)^47^ as covariates.

For the first hypothesis, we used the sample of *N* = 95 individuals per group and used two linear mixed effects models with methylation as dependent variable and group as predictor. We entered random intercepts for individual and CpG unit. In the second model, we then entered absolutes of cell type abundance as covariates. Abundance of cell types was z-standardized.

In the longitudinal analyses we were interested in treatment responsive CpG units. Therefore, all CpG units were included in the following analyses, while we controlled for nicotine consumption and medication effects by including covariates in the models used.

For our second hypothesis, we used a linear mixed effects model (LMM) with methylation as dependent variable, time, group and their interaction as fixed effects with cell types, nicotine consumption and medication as covariates. As the LMM with random intercept and slope for CpG units did not converge in this analysis, we averaged methylation across loci to obtain a subject-level methylation estimate, resulting in a single mean methylation value per participant and measurement point. We entered uncorrelated random intercepts and slopes for individual. Abundance of covariates was z-standardized.

Then, we assessed whether depression severity decreased in the group of patients over a two-week treatment period with a paired t-test. To obtain a subject-level methylation estimate, CpG-specific methylation differences were averaged across loci, resulting in a single mean methylation difference per participant (Δmethylation). Change in depression severity Δdepression as well as change in cell type composition (Δabsolute abundance) was calculated as the difference between the two measurement points (t0-t1). Next, we performed linear regression with Δmethylation as dependent variable and Δdepression severity as well as group and the interaction of both as predictors. In addition, Δabsolute abundance of neutrophilic granulocytes, lymphocytes, basophiles, eosinophiles and monocytes (t0-t1) as well as nicotine consumption and dose equivalents of antidepressants and neuroleptics were included as covariates. All metric predictors were z-standardized.

To analyze the associations of methylation and mRNA at t0 (fourth hypothesis) we used a LMM. Methylation was entered as dependent variable, mRNA abundance and group and their interaction as predictors. Random intercepts for individuals and CpG-units were entered in the model. All metric predictors were z-standardized. In addition, to facilitate comparison of the associations of single CpG sites with mRNA abundance between our study and other/future studies, we provide exploratory pairwise Pearson-correlation coefficients for single CpG sites of the three genes with mRNA abundance of the respective genes for patients and controls. This is also in line with notions that transcriptional activity may be influenced by specific CpG sites at regulatory regions rather than global methylation of a gene^48^. P-values of these analyses were adjusted for false discovery rate (FDR).

**Table 1:** Descriptive statistics and Welch’s t-tests after propensity score matching.

Variable	HC			MDD			t	df	*p*	d
	Mean	SD	*n*	Mean	SD	*n*				
Sample for cross-sectional analyses										
Age	39.86	14.03	95	42.06	13.95	95	-1.08	188.0	0.280	0.16
Nicotine	1.12	3.91	95	6.72	8.57	95	-5.79	131.5	< 0.001	0.84
Alcohol	4.56	6.56	95	3.39	11.46	95	0.86	149.7	0.390	-0.13
BMI	24.84	5.03	95	26.43	6.20	95	-1.93	180.4	0.055	0.21
Age of onset				25.68	13.31	87				
Number of episodes				5.76	9.06	78				
Number of inpatient treatments				1.99	1.54	87				
Sample for longitudinal analyses										
Age	43.58	13.45	66	44.82	12.82	66	-0.54	129.7	0.588	0.10
Nicotine	1.46	4.50	66	5.53	7.39	66	-3.83	107.4	< 0.001	0.67
Alcohol	4.63	7.17	66	5.35	24.00	66	-0.23	76.5	0.817	0.00
BMI	25.82	4.41	66	27.71	6.64	66	-1.93	113.1	0.057	0.34
Age of onset				27.49	13.71	65				
Number of episodes				5.43	8.31	58				
Number of inpatient treatments				2.00	1.63	63				

Age in years. Nicotine in cigarettes/day. Alcohol in grams/day. BMI in kg/m^2^. Age of onset in years.

## Results

### Group differences in DNA-methylation at the first measurement point

It is important to mention that data overlapped with data of a previous study^15^ for this analysis. There was no significant group difference in either *SLC6A4* or *NR3C1* methylation and group explained no variance in methylation of either gene (*SLC6A4*: marginal *R*^*2*^ = 0.00, conditional *R*^*2*^ = 0.66; *NR3C1*: marginal *R*^*2*^ = 0.00, conditional *R*^*2*^ = 0.86). As expected and shown in our previous studies^16,49^ there was a significant group difference in *OXT* methylation with patients having lower *OXT* methylation levels (Table 2). This effect explained a small amount of variance in *OXT* methylation (marginal *R*^*2*^ = 0.01, conditional *R*^*2*^ = 0.90) and should be interpreted with caution since the bootstrapped CI contained zero.

Entering abundance of celltypes as covariates revealed significant effects of both cell types on *OXT* methylation, while group was not a significant predictor anymore. Neutrophilic granulocytes were negatively associated with *OXT* methylation, while there was a positive association between lymphocytes and *OXT* methylation. Fixed effects explained a small amount of variance in *OXT* methylation in the model containing cell type abundance (marginal *R*^*2*^ = 0.03, conditional *R*^*2*^ = 0.89). The bootstrapped CIs of the effects of neutrophilic granulocyte abundance as well as lymphocyte abundance contained zero, these effects should be interpreted with caution.

**Table 2:** Fixed effects of the LMMs testing group differences at the first measurement point with respective dependent variables, bootstrapped standardized CIs (iterations = 5000) and standardized estimates with *p*-values.

Predictors	*Estimates*	*CI*	*p*	Predictors	*Estimates*	*CI*	*p*
Dependent variable: *SLC6A4* methylation							
(Intercept)	0.10	0.05–0.14	< 0.001	(Intercept)	0.10	0.04–0.15	< 0.001
Group	0.00	−0.01–0.01	0.977	NG	-0.00	−0.01–0.01	0.662
				Lymphocytes	0.01	−0.00–0.01	0.007
				Basophiles	-0.00	−0.01–0.01	0.633
				Eosinophiles	-0.00	−0.01–0.01	0.154
				Monocytes	-0.00	−0.01–0.01	0.570
				Group	0.01	−0.01–0.02	0.085
Dependent variable: *NR3C1* methylation							
(Intercept)	0.06	0.01–0.12	< 0.001	(Intercept)	0.06	0.00–0.12	< 0.001
Group	0.00	−0.00–0.01	0.094	NG	0.00	−0.01–0.01	0.745
				Lymphocytes	-0.00	−0.01–0.01	0.598
				Basophiles	0.00	−0.01–0.01	0.809
				Eosinophiles	-0.00	−0.01–0.01	0.910
				Monocytes	-0.00	−0.01– 0.01	0.630
				Group	0.00	−0.01–0.01	0.810
Dependent variable: *OXT* methylation							
(Intercept)	0.42	0.16–0.61	< 0.001	(Intercept)	0.43	0.24–0.62	< 0.001
Group	-0.03	-0.08–0.02	0.026	NG	-0.03	−0.07–0.02	0.003
				Lymphocytes	0.03	−0.01–0.07	0.001
				Basophiles	-0.01	−0.05–0.02	0.190
				Eosinophiles	-0.00	−0.04–0.03	0.799
				Monocytes	-0.00	−0.04–0.04	0.810
				Group	-0.00	−0.06–0.06	0.818

NG = neutrophilic granulocytes. Reference level for group is healthy control. Left column: effect of group on methylation. Right column: Effect of group on methylation controlled for cell type abundance.

### Group differences of changes in DNA-methylation over the two-week treatment period

There was a significant interaction between group and time for SLC6A4 methylation. This interaction is due to a slight increase in HC and a slight decrease in methylation in the MDD group. As shown in the cross-sectional analysis, lymphocytes were a significant predictor of *OXT*-methylation. It seems that differences in the abundance of cell types between patients and controls explain differences in *OXT* methylation, i.e., *OXT*-methylation is positively associated with lymphocytes. In addition, there was a significant effect of time on OXT methylation and a significant interaction between time and group. Methylation increased over time and more so in controls as compared to MDD patients. However, bootstrapped CIs contained zero for all significant fixed effects predicting *SLC6A4* or *NR3C1* methylation as well as for the interaction effect in the *OXT* model. These effects should be interpreted with caution (Table 3).

**Table 3:** Fixed effects of the models with respective dependent variables, bootstrapped standardized CIs (iterations = 5000) and standardized estimates with *p*-values.

Predictors	*Estimates*	*CI*	*p*
Dependent variable: *SLC6A4* methylation			
(Intercept)	0.10	0.09–0.12	< 0.001
NG	0.00	−0.01–0.01	0.884
Lymphocytes	0.00	−0.01–0.01	0.672
Basophiles	0.00	−0.01–0.01	0.111
Eosinophiles	0.00	−0.01–0.01	0.790
Monocytes	−0.00	−0.01–0.00	0.090
Nicotine	0.00	−0.01–0.01	0.381
DE antidepressants	−0.00	−0.01–0.01	0.871
DE neuroleptics	−0.00	−0.01–0.01	0.680
Group	0.01	−0.02–0.03	0.438
Time	0.01	−0.01–0.03	0.012
Group × Time	−0.02	−0.05–0.01	0.015
Dependent variable: *NR3C1* methylation			
(Intercept)	0.09	0.08–0.10	< 0.001
NG	−0.00	−0.01–0.01	0.970
Lymphocytes	−0.00	−0.01–0.00	0.768
Basophiles	−0.00	−0.01–0.00	0.753
Eosinophiles	0.00	−0.00–0.01	0.745
Monocytes	−0.00	−0.01–0.00	0.282
Nicotine	0.00	−0.01–0.00	0.974
DE antidepressants	0.00	−0.00–0.01	0.396
DE neuroleptics	−0.00	−0.01–0.00	0.046
Group	0.00	−0.01–0.02	0.542
Time	−0.00	−0.01–0.01	0.399
Group × Time	0.01	−0.01–0.02	0.104
Dependent variable: *OXT* methylation			
(Intercept)	0.38	0.33–0.43	< 0.001
NG	−0.01	−0.04–0.02	0.127
Lymphocytes	0.03	−0.01–0.05	0.001
Basophiles	0.00	−0.03–0.03	0.627
Eosinophiles	−0.01	−0.03–0.01	0.133
Monocytes	−0.01	−0.05–0.02	0.196
Nicotine	0.00	−0.03–0.03	0.808
DE antidepressants	−0.02	−0.06–0.03	0.186
DE neuroleptics	0.01	−0.03–0.03	0.520
Group	0.03	−0.06–0.11	0.297
Time	0.04	0.00–0.09	< 0.001
Group × Time	−0.04	−0.10–0.02	0.017

NG = neutrophilic granulocytes; DE = dose equivalents; Nicotine in cigarettes/day. Reference level for group is healthy control.

### Associations between change in depression severity and change in DNA-methylation

Depression severity decreased significantly in the group of patients suffering from depression after a two-week treatment period (t0: *M* = 28.88, *SD* = 12.47; t1: *M* = 20.94, *SD* = 12.87; *t*(62) = 7.53, *p* < .001, *d* = 0.95). Regarding change in DNA-methylation, there were neither significant main effects of change in depression severity or group nor significant interactions between change in depression severity and group on either of the three genes (Table 4). Neither of the three models explained significantly more variance in methylation of the respective gene than the nullmodel (SLC6A4: *F(11*,*75)* = 0.83, *p* = .61; NR3C1: *F(11*,*75)* = 0.89, *p* = .55; OXT: *F(11*,*69)* = 1.06, *p* = .41). Thus, changes in methylation of the three target genes were not associated with changes in depression severity over a two-week period.

**Table 4:** Linear regression models with difference in methylation of the respective gene as dependent variable.

Variable	beta (SE)	t	*p*
*SLC6A4*			
ΔNG	0.00 (0.00)	−0.42	0.678
ΔLymphocytes	0.00 (0.00)	−0.25	0.807
ΔBasophiles	−0.01 (0.00)	−2.43	0.017
ΔEosinophiles	0.00 (0.00)	0.02	0.984
ΔMonocytes	0.00 (0.00)	0.60	0.550
Nicotine	0.00 (0.00)	0.37	0.716
DE antidepressants	0.00 (0.00)	−0.24	0.814
DE neuroleptics	0.00 (0.00)	0.43	0.668
ΔBDI-II	−0.01 (0.01)	−0.44	0.659
Group	0.00 (0.01)	−0.01	0.994
Group*ΔBDI-II	0.00 (0.01)	0.63	0.530
*NR3C1*			
ΔNG	0.00 (0.00)	0.25	0.807
ΔLymphocytes	0.00 (0.00)	−0.16	0.869
ΔBasophiles	0.00 (0.00)	1.24	0.220
ΔEosinophiles	0.00 (0.00)	−0.72	0.474
ΔMonocytes	0.00 (0.00)	−0.15	0.882
Nicotine	0.00 (0.00)	0.79	0.430
DE antidepressants	0.00 (0.00)	1.45	0.151
DE neuroleptics	0.00 (0.00)	1.39	0.169
ΔBDI-II	0.00 (0.01)	0.03	0.978
Group	0.00 (0.01)	−0.36	0.719
Group*ΔBDI-II	0.00 (0.01)	0.05	0.959
*OXT*			
ΔNG	−0.01 (0.01)	−1.33	0.187
ΔLymphocytes	0.01 (0.01)	0.48	0.634
ΔBasophiles	0.02 (0.01)	1.64	0.105
ΔEosinophiles	−0.01 (0.01)	−0.82	0.415
ΔMonocytes	0.01 (0.01)	1.21	0.231
Nicotine	0.00 (0.01)	0.34	0.732
DE antidepressants	−0.01 (0.01	−0.45	0.653
DE neuroleptics	−0.00 (0.01)	−0.36	0.719
ΔBDI-II	−0.03 (0.03)	−0.97	0.334
Group	−0.04 (0.03)	−1.34	0.185
Group*ΔBDI-II	0.02 (0.02)	0.79	0.431

NG = neutrophilic granulocytes. BDI-II: depression severity. DE: dose equivalents. Nicotine in cigarettes/day. Reference level for group is healthy control.

### Associations between DNA methylation and mRNA abundance

For *SLC6A4* and *OXT* there was no significant association between either of the predictors (mRNA, group and their interaction) and DNA-methylation (Table 5). Since the LMM with *NR3C1* as dependent variable failed to converge across all optimizers, we applied a linear regression using mean *NR3C1* methylation as dependent variable and group, mRNA and their interaction as predictors. This model did not significantly outperform the null model (*F*(3,90) = 2.39, *p* = .07; *R2* = 0.04). Exploratory correlation analyses revealed that only CpG site 5 (chr20:3071505–3071506; *r* = -.42; *p* = .013 FDR adjusted) of OXT was significantly negatively associated with mRNA abundance of *OXT* in healthy controls. Correlation coefficients and FDR adjusted p-values are in the supplement (Table S2).

**Table 5:** Fixed effects of the models with respective dependent variables, standardized estimates, *df*, *t*- and *p*-values.

Variable	*beta (SE)*	*df*	*t*	*p*
Dependent variable: *SLC6A4* methylation				
(Intercept)	0.11 (0.02)	36.7	5.76	0.000
Group	−0.00 (0.01)	118.5	0.01	0.992
mRNA	0.01 (0.00)	118.3	1.24	0.216
Group*mRNA	−0.00 (0.00)	124.9	-0.53	0.594
Dependent variable: *OXT* methylation				
Intercept	0.40 (0.05)	22.5	7.99	0.000
Group	−0.02 (0.02)	111.2	−0.79	0.429
mRNA	−0.01 (0.02)	111.7	−0.46	0.644
Group*mRNA	0.00 (0.02)	111.3	0.08	0.938

Reference level for group is healthy control.

## Discussion

In the present study, we examined group differences in methylation between patients and controls at the beginning of inpatient treatment. Thereafter, we investigated whether initial group differences between patients and controls become smaller after a two-week inpatient treatment and whether a decrease in group differences is associated with a decrease in depression severity in patients. In addition, we investigated whether methylation is associated with mRNA abundance to see if differences in methylation are functionally relevant. Please note that the sample overlapped with the sample of our previous study^15^. All analyses without overlapping samples can be found in the supplement (Tables S3–S7). Results were similar.

As expected and shown in two separate previous studies^15,16^, we found a significant group difference in *OXT* methylation. Patients showed lower methylation levels compared to controls. This group difference in *OXT*-methylation completely vanished when controlling abundance of neutrophilic granulocytes and lymphocytes. Neutrophils were inversely associated with *OXT*-methylation while lymphocytes were positively associated with *OXT*-methylation indicating differential methylation patterns of *OXT* in different blood cell types. The association between cell type abundance and *OXT* methylation indicates that if the absolute amount of a cell type associated with *OXT*-methylation is higher/lower in one group, *OXT*-methylation levels in peripheral blood will be higher/lower as well. If cell types are not controlled, this will result in group differences in *OXT* methylation that could erroneously be interpreted as relevant for depression development or treatment. We did not find group differences in *SLC6A4* methylation. This is in line with current meta-analyses reporting heterogeneous findings for group differences in methylation of *SLC6A4* methylation^7^. This is also in line with results of EWAS studies: These studies mostly found differentially methylated regions in genes that have not been examined in target gene studies, e.g. genes associated with immune response or neural development, since their association with depression is not straightforward before the background of theories of depression development^50,51^. Thus, accumulating epigenetic evidence indicates that depression is less characterized by alterations in a few brain-related target genes than by system-wide abnormalities. This supports the view of depression as a systemic disorder that manifests in somatic and psychological symptoms.

We found significant interactions of group and time for *SLC6A4* and *OXT*. There was a small increase in methylation in the control group and a decrease (*SLC6A4*)/nearly no change (*OXT*) in the group of inpatients. However, bootstrapped CIs of these small interaction effects contained zero, so that this effect should be interpreted with caution. Therefore, these changes might not be associated with inpatient treatment. Since there were no significant group differences in methylation after controlling for cell type abundance at the first measurement point, our initial hypothesis of decreasing group differences with treatment response had to be rejected.

While depression severity significantly decreased after a two-week treatment period, we did not find significant associations between change in DNA-methylation and changes in depression severity. However, the idea that methylation could be a key mechanism in early treatment response is still promising. While most studies on changes of DNA-methylation of *NR3C1* and *SLC6A4* showed changes across longer time scales, i.e., months to years^52–54^, there are also studies showing methylation changes of genes (*OXTR*, *PGC-1α*, *PDK4*, *PPARδ*) within minutes or days after stress and physical activity^55–58^– both relevant factors in depression development and treatment. Furthermore, some studies found an effect of psychotherapeutic interventions on methylation of the examined DNA-regions – which was mostly investigated for PTSD treatment−^30,53^, while there are also studies that did not find DNA-methylation changes of common target genes following psychotherapy for PTSD, when rigorously controlling cell type composition^59^. For MDD there are some studies with less than 50 patients and one study with *n* = 221 patients investigating response to treatment with SSRIs, electroconvulsive therapy (ECT) and trauma-focused therapy for MDD^31,60–62^. Two studies found significant decreases in *NR3C1* methylation after treatment with ECT and SSRIs respectively^60,62^. In the study investigating ECT, *NR3C1* methylation was lower in patients before the treatment and decreased further especially in treatment responders^60^. Mohammadi and colleagues^62^ also found a decrease in *SLC6A4* methylation after 100 days of SSRI treatment. As we aimed to investigate whether initial treatment response is associated with changes in DNA-methylation, our study utilized a two-week interval between measurements. While we observed overall changes in depression severity, a substantial proportion of patients does not show significant improvement within this timeframe. If DNA-methylation accounts for a portion of the variance in depression severity, a two-week observation period may be insufficient to capture biological changes with an effect sizes much smaller than the one for change in depression severity. On the other hand, the absence of short-term methylation changes in our study does not necessarily indicate stability but may instead reflect a delayed or downstream involvement of these loci. The largest study, that we know of, found that three CpG sites in *SLC6A4* increased or decreased their methylation after six weeks of SSRI treatment^61^. The change in methylation was not associated with symptom reduction or any other variable of depression course. This study is in line with our results, since we also did not observe a systematic change in *SLC6A4* methylation. Therefore, even though our data does not suggest that these three target genes are biomarkers of early treatment effects, there could be other genes that are dynamically methylated and cause early treatment response. The study by Carvalho Silva and colleagues^31^ for example found methylation changes in genes associated with inflammatory processes and the tumor necrosis factor signaling pathway.

Our study also highlights the main issue that research on epigenetics in psychiatry faces: Methylation can only be measured in peripheral organic material in alive humans. The combination of confounding factors of peripheral tissues as seen in our study and the incomplete understanding of the interplay between methylation of peripheral and central tissues complicate the interpretation of current epigenetic research in psychiatry. Even though there have been attempts to shed light on the associations between methylation of central tissues and depression as well as the associations between central and peripheral methylation in post-mortem studies, there are still hindrances regarding sample size, the potentially dynamic nature of central and peripheral methylation, their retrospective nature regarding the associations between psychiatric conditions and biological variables as well as the impossibility of longitudinal analyses of central methylation change with symptom course. Recent findings of post-mortem studies suggest low to moderate associations between central and peripheral tissue with considerable tissue-specific variance^63,64^. This becomes even more complicated as there also seems to be region and cell-type specific methylation in the brain, too^65^. There are attempts to address these issues, e.g., with a single cell methylome atlas of the brain^66^. However, even though these attempts seem promising, their usefulness for the investigation of methylation biomarkers for depression has yet to be shown. In summary, our study highlights that research on epigenetic biomarkers for depression development and course is complicated by methodological issues and that epigenetic correlates of peripheral tissues must be interpreted with caution.

We did not find associations, i.e., main effects of mRNA abundance or an interaction with group, when examining the associations between mRNA abundance of *SLC6A4*-, *NR3C1*- or *OXT*- and methylation of these genes. A review on *NR3C1* methylation studies summarizes that studies mostly found an inverse association between *NR3C1*-methylation and mRNA abundance but also mixed results (positive and negative associations) as well as nullfindings^67^. *NR3C1* exon 1 F methylation was generally very low across studies^67^ and one study even concludes that no methylation was observed in most of the CpG sites in *NR3C1*^68^. Therefore, there could be other regions of *NR3C1* that have a stronger methylation signature and thus associations with gene transcription more likely to be detected. For *SLC6A4* on the other hand, there are mixed results with CpG site specific associations and moderations by genotype^61,69^. *OXT* methylation and *OXT* mRNA in peripheral tissue have – to the best of our knowledge – not yet been investigated. Therefore, our results could be valuable for future research on the role oxytocin in mental disorders. It is important to note that the lack of peripheral associations between methylation and mRNA abundance in our study does not necessarily transfer to associations between central methylation and mRNA measures. In addition, the associations between methylation and mRNA abundance could be moderated by other (epigenetic) processes like for example histone deacetylation^70^. Further, results between studies could vary as a function of the exact genomic location, i.e., the CpG sites, investigated, differences in covariates, individuals and measurement methods for mRNA abundance^71^. Our finding that methylation of one CpG site in *OXT* is significantly negatively associated with mRNA abundance in healthy controls suggests that transcriptional activity could be regulated by specific sites rather than overall methylation status of the promoter region of *OXT*.

There are some limitations that need to be considered when interpreting the results of our study. First, our sample size allowed us to detect small to medium sized interaction effects of time and group with a power of 80%. However, there still could be small group specific changes of methylation over time even after controlling covariates. Second, we investigated peripheral blood. Other peripheral tissues, e.g., saliva samples, could have fewer confounding factors and be easier to interpret regarding associations between methylation and depression. Third, we used a naturalistic design with two measurement points separated by two weeks. While ecologically valid, more measurement points and a longer observation period as well as standardization and comparison of treatments could paint a more nuanced picture of the associations between DNA-methylation in peripheral tissue and depression treatment and course. Standardization of treatment could also shed light on the mechanisms responsible for changes in DNA methylation.

In conclusion, we did not find associations between *NR3C1*, *SLC6A4* or *OXT* methylation status and depression diagnosis or change of depressive symptoms. While SLC6A4 and OXT methylation were significantly changing over a two-week treatment period as a function of group, these effects were small and bootstrapped CIs for interactions with group contained zero. Therefore, these changes might not be associated with inpatient treatment. Regarding change in depression severity, we found a significant decrease after the two-week treatment period in patients. Further, we found significant associations between methylation of *OXT* and the abundance of neutrophils and lymphocytes. Therefore, the previously published hypomethylation of *OXT* in patients suffering from depression compared to controls^16,49^ seems to be a result of elevated white blood cells in patients. There was no overall association between *NR3C1*, *OXT* or *SLC6A4* methylation and mRNA abundance but a negative association between a single CpG site in *OXT* and mRNA abundance of *OXT* in healthy controls. Our study highlights the importance of including confounding factors when investigating associations of mental disorders and methylation in peripheral blood samples, which should be rigorously controlled in future research. Progress in the field will require large, longitudinal studies integrating precise sample characterization and careful adjustment for confounders to ensure reported associations reflect disease-associated mechanisms rather than confounding variables.

## Supplementary Information

Below is the link to the electronic supplementary material.

Supplementary Material 1

## Data Availability

Data will be shared upon reasonable request.
