# Supplementary material for: Longitudinal associations of DNA-methylation of OXT, SLC6A4 and NR3C1 genes with the treatment response in patients with depression
Source: Sci Rep. 2026 Jun 16;16:18709. doi: 10.1038/s41598-026-57641-9 (PMC13272670; doi:10.1038/s41598-026-57641-9)
Supplement: Supplementary file 1 — Supplementary Material 1 [file 41598_2026_57641_MOESM1_ESM.pdf]

Longitudinal associations of DNA-Methylation of OXT, SLC6A4 and NR3C1 genes with the treatment response in patients with depression

Simon Sanwald[1], Thomas Kammer[1], Bernhard J. Connemann[1], Christian Montag[2,3,4] & Markus Kiefer[1]

[1] Department of Psychiatry and Psychotherapy III, Ulm University, Ulm, Germany

[2] Centre for Cognitive and Brain Sciences, Institute of Collaborative Innovation, University Macau, Macau SAR, China

[3]Department of Psychology, Faculty of Social Sciences, University of Macau, Macau SAR, China

[4]Department of Computer and Information Science, Faculty of Science and Technology, University of Macau, Macau SAR, China

Supplementary Material

Characteteristics of the inpatient sample

Table S1 shows medication classes and other therapeutic approaches administered to inpatients of our longitudinal sample during the two week treatment period.

Table S1.

Therapeutic approaches in the group of MDD inpatients.

**Antidepressants**	***n***	**%**
SSRIs	37	56
SNRIs	16	24
TCAs	13	20
TeCAs	10	15
Atypical	8	12
**Neuroleptics**	***n***	**%**
First generation	11	17
Second generation	1	2
ECT	2	3
Sleep deprivation	2	3
BLT	3	5
Esketamine	2	3
Lithium	1	2
CBT	56	85

Note. SSRI: selective serotonin reuptake inhibitors; SNRI: serotonin-norepinephrine reuptake inhibitors; TCA: tricyclic antidepressants; TeCA: tetracyclic antidepressants; ECT: electroconvulsive therapy; BLT: bright light therapy; CBT: cognitive behavioral therapy.

Correlation Analysis for associations between SLC6A4, NR3C1 and OXT methylation and mRNA abundance

Table S2. Correlation coefficients and FDR adjusted p-values for associations between DNA-methylation and mRNA abundance of the respective genes.

	MDD		HC	
SLC6A4				
CpG	*r*	*p*	*r*	*p*
S_CpG_1	-0.17	0.756	0.04	0.927
S_CpG_3	0.13	0.756	0.03	0.927
S_CpG_4	0.12	0.756	-0.11	0.778
S_CpG_5.6	0.16	0.756	-0.17	0.726
S_CpG_7	-0.06	0.866	0.16	0.726
S_CpG_8.9	-0.05	0.866	-0.04	0.927
S_CpG_10	-0.05	0.866	-0.05	0.927
S_CpG_11-15	0.13	0.756	0.07	0.898

S_CpG_16.17	0.04	0.866	-0.13	0.763
S_CpG_18.19	0.11	0.756	-0.12	0.763
S_CpG_27	-0.06	0.866	0.16	0.726
S_CpG_28	0.27	0.494	-0.04	0.927
S_CpG_29.30	0.03	0.866	-0.02	0.949
S_CpG_31.32	-0.21	0.756	-0.17	0.726
S_CpG_39-42	-0.10	0.854	-0.17	0.726
S_CpG_43.44	0.13	0.756	-0.02	0.949
S_CpG_45	0.00	0.993	-0.04	0.927
S_CpG_46	-0.08	0.866	0.20	0.726
S_CpG_47.48	0.18	0.756	-0.16	0.726
S_CpG_52	-0.17	0.756	0.00	0.999
S_CpG_53.54	-0.04	0.866	0.10	0.858
S_CpG_57.58	-0.03	0.866	-0.31	0.625
S_CpG_60	-0.15	0.756	-0.03	0.927
S_CpG_61-63	-0.07	0.866	-0.10	0.858
S_CpG_64	-0.09	0.866	-0.24	0.726
S_CpG_65	-0.32	0.494	0.10	0.858
S_CpG_66	-0.13	0.756	-0.23	0.726
S_CpG_68	-0.17	0.756	-0.01	0.968
S_CpG_69	-0.15	0.756	-0.08	0.898
S_CpG_70.71	-0.29	0.494	-0.15	0.726
NR3C1				
N_CpG_1.2	0.00	0.985	0.00	0.978
N_CpG_3.4.5	0.00	0.985	0.02	0.978
N_CpG_9	0.10	0.867	-0.04	0.978
N_CpG_10.11	-0.10	0.867	0.01	0.978
N_CpG_12.13	0.23	0.662	-0.03	0.978
N_CpG_14	-0.18	0.697	0.03	0.978
N_CpG_17.18	0.05	0.985	-0.13	0.978
N_CpG_20.21	0.24	0.662	0.06	0.978
N_CpG_26	-0.09	0.867	0.00	0.978
N_CpG_35	-0.02	0.985	0.16	0.978
N_CpG_36	-0.22	0.662	0.32	0.164
N_CpG_38.39	-0.15	0.827	0.03	0.978
N_rev_CpG_6.7	-0.10	0.867	0.28	0.185
N_rev_CpG_8	0.03	0.985	-0.08	0.978
N_rev_CpG_10.11	-0.02	0.985	-0.06	0.978
N_rev_CpG_12.13	-0.18	0.697	-0.03	0.978
OXT				
O_CpG_1.2	-0.11	0.853	-0.12	0.413
O_CpG_3	-0.18	0.794	-0.15	0.389
O_CpG_4	-0.06	0.853	-0.33	0.055
O_CpG_5	-0.02	0.963	-0.42	0.013
O_CpG_7.8	-0.02	0.963	-0.16	0.389
O_CpG_9.10	-0.09	0.853	-0.21	0.260
O_CpG_13	-0.17	0.794	-0.29	0.081

O_CpG_14.15	-0.10	0.853	-0.14	0.389
O_CpG_16	0.00	0.980	-0.03	0.797
O_CpG_18	-0.33	0.357	-0.25	0.200
O_CpG_19	-0.07	0.853	-0.14	0.413
O_CpG_20	-0.44	0.598	-0.19	0.389
O_CpG_24.25	-0.14	0.853	-0.13	0.413
O_CpG_26	-0.08	0.853	-0.08	0.588

Note. MDD: patients with Major Depressive Disorder; HC: healthy controls.

Analysis of group differences at t0

In the cross-sectional sample, inclusion of all CpG sites and controlling for nicotine and medication resulted in similar results as compared to the analyses provided in the main article, in which CpG sites associated with nicotine and medication were excluded. Lymphocyte abundance was a significant predictor of SLC6A4 methylation. Group was not a significant predictor of OXT methylation, while inclusion of celltypes revealed a significant association between neutrophilic granulocytes as well as lymphocytes and OXT methylation. Group was a significant predictor of NR3C1 methylation. The group difference in NR3C1 methylation was not significant when including celltypes as covariates. Instead, dose equivalents of neuroleptics were significant. All effects should be interpreted with caution since all bootstrapped CIs contained zero (Table S2).

All additional analyses in the longitudinal sample without patients analyzed in our previous study showed a similar result pattern as compared to the results presented in our main article.

Table S3. Fixed effects of the LMMs testing group differences at the first measurement point with respective dependent variables, bootstrapped standardized CIs (iterations = 5000) and standardized estimates with *p*-values.

Dependent variable: SLC6A4 methylation							
Predictors	*Estimates*	*CI*	*p*	Predictors	*Estimates*	*CI*	*p*
(Intercept)	0.11	0.03 – 0.17	<0.001	(Intercept)	0.11	0.04 – 0.17	<0.001
Nicotine	0.00	-0.01 – 0.01	0.589	NG	-0.00	-0.01 – 0.01	0.858
DE antidepressants	0.00	-0.01 – 0.01	0.478	Lymphocytes	0.00	-0.00 – 0.01	0.037
DE neuroleptics	-0.00	-0.01 – 0.00	0.216	Basophiles	-0.00	-0.01 – 0.01	0.705
Group	-0.00	-0.02 – 0.02	0.750	Eosinophiles	-0.00	-0.01 – 0.01	0.605
				Monocytes	-0.00	-0.01 – 0.01	0.455
				Nicotine	0.00	-0.01 – 0.01	0.155
				DE antidepressants	0.00	-0.01 – 0.01	0.767
				DE neuroleptics	-0.00	-0.01 – 0.00	0.109
				Group	0.01	-0.02 – 0.03	0.421
Dependent variable: NR3C1 methylation							
Predictors	*Estimates*	*CI*	*p*	Predictors	*Estimates*	*CI*	*p*
(Intercept)	0.08	-0.07 – 0.21	0.025	(Intercept)	0.09	-0.05 – 0.22	0.016
Nicotine	-0.00	-0.01 – 0.00	0.278	NG	-0.00	-0.01 – 0.01	0.763
DE antidepressants	-0.00	-0.01 – 0.01	0.590	Lymphocytes	-0.00	-0.01 – 0.01	0.866

DE neuroleptics	-0.00	-0.01 – 0.00	0.104	Basophiles	0.00	-0.00 – 0.01	0.336
Group	0.01	-0.00 – 0.02	0.009	Eosinophiles	0.00	-0.01 – 0.01	0.784
				Monocytes	-0.00	-0.01 – 0.00	0.213
				Nicotine	-0.00	-0.01 – 0.01	0.366
				DE antidepressants	-0.00	-0.01 – 0.01	0.978
				DE neuroleptics	-0.00	-0.01 – 0.00	0.010
				Group	0.01	-0.01 – 0.02	0.119

Dependent variable: *OXT* methylation							
Predictors	*Estimates*	*CI*	*p*	Predictors	*Estimates*	*CI*	*p*
(Intercept)	0.38	0.23 – 0.54	<0.001	(Intercept)	0.38	0.22 – 0.57	<0.001
Nicotine	-0.01	-0.04 – 0.02	0.246	NG	-0.02	-0.06 – 0.01	0.028
DE antidepressants	-0.01	-0.05 – 0.02	0.193	Lymphocytes	0.04	-0.00 – 0.07	0.001
DE neuroleptics	-0.01	-0.04 – 0.02	0.481	Basophiles	-0.01	-0.04 – 0.02	0.197
Group	0.01	-0.08 – 0.09	0.798	Eosinophiles	-0.00	-0.04 – 0.03	0.721
				Monocytes	-0.01	-0.04 – 0.03	0.329
				Nicotine	-0.00	-0.04 – 0.04	0.799
				DE antidepressants	-0.01	-0.06 – 0.04	0.255
				DE neuroleptics	-0.00	-0.03 – 0.03	0.710
				Group	0.03	-0.06 – 0.12	0.166

Note. NG = neutrophilic granulocytes; DE = dose equivalents. Reference level for group is healthy control. Left column: effect of group on methylation. Right column: Effect of group on methylation controlled for cell type abundance. Estimates and CIs standardized.

Table S4. Fixed effects of the LMMs testing group differences at the first measurement point with respective dependent variables, bootstrapped standardized CIs (iterations = 5000) and standardized estimates with *p*-values for a sample without previously analyzed patients.

Dependent variable: SLC6A4 methylation							
Predictors	*Estimates*	*CI*	*p*	Predictors	*Estimates*	*CI*	*p*
(Intercept)	0.12	0.04 – 0.20	<0.001	(Intercept)	0.11	0.03 – 0.20	<0.001
Nicotine	0.00	-0.01 – 0.01	0.362	NG	0.00	-0.01 – 0.02	0.443
DE antidepressants	0.00	-0.01 – 0.02	0.466	Lymphocytes	0.00	-0.01 – 0.01	0.808
DE neuroleptics	-0.00	-0.01 – 0.01	0.470	Basophiles	0.00	-0.01 – 0.02	0.727
Group	-0.00	-0.03 – 0.02	0.806	Eosinophiles	-0.00	-0.02 – 0.01	0.458
				Monocytes	-0.01	-0.02 – 0.01	0.188
				Nicotine	0.00	-0.01 – 0.01	0.470
				DE antidepressants	0.00	-0.01 – 0.01	0.983
				DE neuroleptics	-0.00	-0.02 – 0.01	0.174
				Group	0.01	-0.02 – 0.04	0.374

Dependent variable: NR3C1 methylation							
Predictors	*Estimates*	*CI*	*p*	Predictors	*Estimates*	*CI*	*p*
(Intercept)	0.05	0.01 – 0.09	<0.001	(Intercept)	0.05	0.01 – 0.10	<0.001

Nicotine	-0.00	-0.01 – 0.00	0.114	NG	0.00	-0.00 – 0.01	0.259
DE antidepressants	-0.00	-0.01 – 0.01	0.753	Lymphocytes	0.00	-0.01 – 0.01	0.817
DE neuroleptics	-0.00	-0.01 – 0.00	0.086	Basophiles	-0.00	-0.01 – 0.01	0.673
Group	0.00	-0.01 – 0.02	0.318	Eosinophiles	0.00	-0.01 – 0.01	0.590
				Monocytes	-0.00	-0.01 – 0.01	0.802
				Nicotine	-0.00	-0.01 – 0.00	0.059
				DE antidepressants	-0.00	-0.01 – 0.01	0.515
				DE neuroleptics	-0.00	-0.01 – 0.00	0.124
				Group	0.00	-0.01 – 0.02	0.461
Dependent variable: *OXT* methylation							
Predictors	*Estimates*	*CI*	*p*	Predictors	*Estimates*	*CI*	*p*
(Intercept)	0.46	0.19 – 0.71	<0.001	(Intercept)	0.46	0.18 – 0.70	<0.001
Nicotine	0.01	-0.03 – 0.04	0.279	NG	-0.01	-0.06 – 0.04	0.313
DE antidepressants	-0.02	-0.06 – 0.03	0.186	Lymphocytes	0.03	-0.01 – 0.08	0.004
DE neuroleptics	0.01	-0.02 – 0.05	0.255	Basophiles	-0.01	-0.06 – 0.05	0.639
Group	-0.01	-0.12 – 0.08	0.615	Eosinophiles	-0.01	-0.06 – 0.05	0.607
				Monocytes	-0.02	-0.07 – 0.02	0.093
				Nicotine	0.02	-0.03 – 0.06	0.212
				DE antidepressants	-0.02	-0.07 – 0.04	0.106
				DE neuroleptics	0.01	-0.02 – 0.05	0.298
				Group	0.01	-0.11 – 0.11	0.866

Note. NG = neutrophilic granulocytes; DE = dose equivalents. Reference level for group is healthy control. Left column: effect of group on methylation. Right column: Effect of group on methylation controlled for cell type abundance. Estimates and CIs standardized.

Table S5. Fixed effects of the models with respective dependent variables, bootstrapped standardized CIs (iterations = 5000) and standardized estimates with *p*-values without previously analyzed patients.

Dependent variable: *SLC6A4* methylation			
Predictors	*Std. Estimates*	*Std. CI*	*p*
(Intercept)	0.11	0.09 – 0.13	<0.001
NG	0.00	-0.01 – 0.01	0.922
Lymphocytes	0.00	-0.01 – 0.02	0.119
Basophiles	0.01	-0.01 – 0.02	0.073
Eosinophiles	-0.01	-0.02 – 0.01	0.075
Monocytes	-0.01	-0.02 – 0.01	0.051
Nicotine	0.00	-0.01 – 0.02	0.144
DE antidepressants	0.00	-0.01 – 0.01	0.827
DE neuroleptics	-0.00	-0.01 – 0.01	0.256
Group	0.00	-0.03 – 0.03	0.944
Time	0.02	-0.00 – 0.04	0.004
Group × Time	-0.03	-0.07 – 0.00	0.001
Dependent variable: *NR3C1* methylation			
(Intercept)	0.09	0.08 – 0.10	<0.001

NG	0.00	-0.01 – 0.01	0.854
Lymphocytes	-0.00	-0.01 – 0.01	0.991
Basophiles	0.00	-0.01 – 0.01	0.990
Eosinophiles	-0.00	-0.01 – 0.01	0.725
Monocytes	0.00	-0.01 – 0.01	0.804
Nicotine	-0.00	-0.01 – 0.00	0.630
DE antidepressants	0.00	-0.01 – 0.01	0.688
DE neuroleptics	-0.00	-0.01 – 0.00	0.156
Group	0.00	-0.02 – 0.02	0.302
Time	-0.00	-0.02 – 0.01	0.649
Group × Time	0.01	-0.01 – 0.02	0.290
Dependent variable: *OXT* methylation			
(Intercept)	0.42	0.36 – 0.45	<0.001
NG	-0.01	-0.04 – 0.02	0.230
Lymphocytes	0.02	-0.01 – 0.06	0.007
Basophiles	0.00	-0.04 – 0.04	0.671
Eosinophiles	-0.01	-0.04 – 0.02	0.232
Monocytes	-0.02	-0.05 – 0.02	0.090
Nicotine	0.01	-0.03 – 0.05	0.453
DE antidepressants	-0.02	-0.06 – 0.02	0.071
DE neuroleptics	0.00	-0.03 – 0.04	0.623
Group	0.01	-0.10 – 0.11	0.661
Time	0.00	-0.04 – 0.05	0.760
Group × Time	-0.01	-0.09 – 0.06	0.562

Note. NG = neutrophilic granulocytes; DE = dose equivalents; Nicotine in cigarettes/day. Reference level for group is healthy control.

Table S6. Linear regression models with difference in methylation of the respective gene as dependent variable without previously analyzed patients.

variable	*beta (SE)*	*t*	*p*
SLC6A4			
ΔNeutrophilic granulocytes	-0.01 (0.00)	-1.95	0.057
ΔLymphocytes	-0.00 (0.00)	-0.64	0.525
ΔBasophiles	-0.01 (0.00)	-1.68	0.098
ΔEosinophiles	-0.00 (0.00)	-1.22	0.228
ΔMonocytes	0.00 (0.00)	0.94	0.351
Nicotine	0.00 (0.00)	0.78	0.440
DE antidepressants	-0.00 (0.00)	-0.03	0.977
DE neuroleptics	0.00 (0.00)	0.55	0.588
ΔBDI-II	-0.01 (0.01)	-0.45	0.655
Group	-0.01 (0.01)	-0.84	0.404
Group*ΔBDI-II	0.00 (0.01)	0.51	0.609
NR3C1			
ΔNeutrophilic granulocytes	0.00 (0.00)	0.44	0.661

ΔLymphocytes	0.00 (0.00)	0.48	0.636
ΔBasophiles	0.01 (0.00)	1.67	0.100
ΔEosinophiles	0.00 (0.00)	0.22	0.827
ΔMonocytes	-0.00 (0.00)	-0.82	0.414
Nicotine	0.00 (0.00)	1.05	0.299
DE antidepressants	0.01 (0.00)	1.49	0.142
DE neuroleptics	0.00 (0.00)	0.15	0.879
ΔBDI-II	0.01 (0.01)	1.24	0.222
Group	0.01 (0.01)	-0.77	0.443
Group*ΔBDI-II	0.01 (0.01)	-0.87	0.389
OXT			
ΔNeutrophilic granulocytes	-0.01 (0.01)	-1.31	0.197
ΔLymphocytes	0.01 (0.01)	0.47	0.638
ΔBasophiles	0.01 (0.01)	1.27	0.210
ΔEosinophiles	-0.00 (0.01)	-0.07	0.946
ΔMonocytes	-0.01 (0.01)	-0.75	0.460
Nicotine	0.00 (0.01)	0.07	0.944
DE antidepressants	-0.01 (0.01	-0.59	0.561
DE neuroleptics	0.00 (0.01)	0.26	0.796
ΔBDI-II	-0.02 (0.03)	-0.61	0.544
Group	-0.01 (0.03)	-0.25	0.805
Group*ΔBDI-II	0.01 (0.02)	0.37	0.710

Note. BDI-II: depression severity.

Regarding the associations between DNA-methylation and mRNA abundance, since the LMM with *NR3C1* as dependent variable failed to converge across all optimizers, we applied a linear regression using mean *NR3C1* methylation as dependent variable and group, mRNA and their interaction as predictors. This model did not significantly outperform the null model ($F(3,58) = 1.63$, $p = .19$; $R2 = 0.03$).

Table S7. Fixed effects of the models with respective dependent variables, standardized estimates, *df*, *t*- and *p*-values without previously analyzed patients.

Dependent variable: *SLC6A4* methylation				
variable	*beta (SE)*	*df*	*t*	*p*
Intercept	0.10 (0.02)	39.9	5.43	0.000
Group	0.00 (0.01)	73.9	0.65	0.515
mRNA	0.01 (0.00)	76.7	1.58	0.119
Group*mRNA	-0.00 (0.00)	75.4	-0.77	0.443
Dependent variable: *OXT* methylation				
Intercept	0.42 (0.06)	31.0	7.38	0.000
Group	-0.04 (0.04)	70.5	-1.11	0.272
mRNA	-0.01 (0.02)	70.9	-0.28	0.782
Group*mRNA	0.02 (0.02)	70.6	1.12	0.266

Note. NG = neutrophilic granulocytes. Reference level for group is healthy control.
